# Medical News and Miscellany

**Published:** 1898-05

**Authors:** 


### Medical News and Miscellany.

**For Sale,** very cheap, a Nedofik operating sofa, the same as used by Dr. Wyeth and in the Polyclinic. Address this office.

**Married,** at Galveston, May 10th (inst.), Dr. Vard H. Hulen to Miss Reba Callaway, daughter of J. M. Callaway, all of Galveston.

**Good practice** and drug store for sale cheap. Good reasons for selling out. Address, P. R., care TEXAS MEDICAL JOURNAL, Austin, Texas.

**The** office of the *Northwestern Medical Journal* has been removed from Minneapolis, Minnesota, to Cedar Rapids, Iowa. Dr. E. N. Fishblatt is editor and proprietor.

**Dr. Andrew Sibley,** formerly of Austin, has located at Palestine. The best wishes of the JOURNAL follow the doctor in his new field of labor and bespeaks for him the success which he so richly merits.

**Errata.**—In printing the cut representing a ruptured abscess of both ovaries, Dr. B. F. Kingsley's communication, page 561, the printer made the mistake of getting the cut upside down. This mistake will, of course, be very evident to our readers.

**Dr. David W. Yandell,** one of the most prominent members of the profession of Louisville, Ky., died in that city on May 2nd. Dr. Yandell was an ex-president of the American Medical Association, and was for a long time senior editor of the *American Practitioner and News.* He was also for many years professor of surgery in the Medical Department of the University of Louisville.

**The New Medical Officers** of the Texas Volunteers are:

First Regiment Texas Volunteer Infantry—Surgeon, Dr. W. N. Vilas, El Paso; assistant surgeon, Dr. William Gammon, Galveston; assistant surgeon, Dr. A. B. Kennedy, Bonham.

Second Regiment—Surgeon, Dr. D. L. Peeples, Navasota; assistant surgeon, Dr. T. T. Jackson, San Antonio; assistant surgeon, Dr. W. B. McLaughlin, Jefferson.

Third Regiment—Surgeon, Dr. H. L. Taylor, Waco; assistant surgeon, Dr. G. W. Sims, Kerrville; assistant surgeon, Dr. W. T. Davidson, Belton.

First Regiment Cavalry—Surgeon, Dr. F. Hadra, San Antonio; assistant surgeon, Dr. H. C. McClanahan, Temple; assistant surgeon, Dr. R. E. Nicholson, Chappel Hill.

---

**One** of the most comfortable and at the same time most economical places in Denver to secure board and the best of accommodations is "The Home," a place especially provided for the care of health seekers. "The Home" is a private place provided with light, airy rooms, abundance of sunshine, good food and the general comforts of home, all at a moderate cost. It is only ten minutes ride by three car lines from the Denver post office, and yet is so situated that one gets a commanding view of the entire city, the plains for hundreds of miles and the Rockies for one hundred and fifty miles, with an uninterrupted view of Pike's Peak, Mt. Evans and Long's Peak. To people from Texas who go to Colorado to spend the summer the management of "The Home" offers rooms which have been thoroughly sterilized and varnished, one person in a room, not by the day but by the week, at $14 per week per person. To reach "The Home," arriving in Denver, any cabman can take you there, or ask for the Sixteenth street cable cars, which will take you (with one transfer) to the very door.

---

**On to Denver.**—The American Medical Association will meet in Denver, June 7th to 10th, inclusive. This meeting bids fair to be one of the largest the Association has ever held. Denver is an ideal place for a convention of this kind; its beautiful location, its mountain scenery, its pure, health-giving atmosphere, and the delightful climate, all combine to make it one of the most attractive places on this continent. The profession and citizens of Denver, we are told, are making the most elaborate provisions for the entertainment of the visitors, and every one is assured of a royal good time. The doctors of Texas are, or should be, especially interested in Colorado, it being one of the nearest and best resorts for a very large class of our health seekers. It is also one of the most delightful places for our citizens to spend the summer, and escape our long hot season. Here the heat is so intense and lasts so long that we contemplate the coming of summer with dread, but in the mountains of Colorado the weather is pleasant the summer through. We wonder that more of our people who can afford it do not take advantage of the magnificent climate and the pure, health-giving atmosphere of Colorado.

